# Ratio of abundances of ciliates behavioral groups as an indicator of the treated wastewater impact on rivers

**DOI:** 10.1371/journal.pone.0275629

**Published:** 2022-10-17

**Authors:** Roman Babko, Volodymyr Pliashechnyk, Jacek Zaburko, Yaroslav Danko, Tatiana Kuzmina, Joanna Czarnota, Joanna Szulżyk-Cieplak, Grzegorz Łagód

**Affiliations:** 1 Department Fauna and Systematics of Invertebrates, National Academy of Sciences of Ukraine, Kyiv, Ukraine; 2 The Municipal Enterprise “Vodokanal of Uzhgorod”, Uzhhorod, Ukraine; 3 Department of Water Supply and Wastewater Disposal, Lublin University of Technology, Lublin, Poland; 4 Department of General Biology and Ecology, Sumy Makarenko State Pedagogical University, Sumy, Ukraine; 5 Department of Ecology and Environmental Protection, Sumy State University, Sumy, Ukraine; 6 Department of Environmental Engineering and Chemistry, Rzeszow University of Technology, Rzeszów, Poland; 7 Department of Technology Fundamentals, Lublin University of Technology, Lublin, Poland; King’s College London, UNITED KINGDOM

## Abstract

A method for assessing the degree of impact of wastewater treatment plant discharge on receiving rivers was proposed, based on the structural indicators of the population of ciliated protozoa. It was shown that the ratio of attached, crawling and free-swimming forms in bottom sediments changes under the influence of discharge. In the points subject to organic pollution, the share of attached filter-feeding bacteriovorous ciliates increases in the assemblage of ciliated protozoa. The proposed Attached Form Index (AFI) takes this ratio into account. The use of AFI makes it possible to assess the restructuring of the assemblage of ciliated protozoa under the influence of point sources of pollution, to establish a zone of negative influence of runoff, to assess the degree of restoration of the aquatic ecosystem, as the influence of the pollution source weakened.

## Introduction

Water bodies are currently exposed to multiple pressures, including dispersed surface runoff and discharges of treated sewage from wastewater treatment plants [1]. Despite the efforts to improve the treatment technology and project financing, many open many water bodies in Europe have significant disturbances in the structure of their biocenoses and a reduced level of biological diversity [2–6]. At the same time, it should be recognized that the European Community pays significant attention to the issues of protection and quality control of the aquatic environment, as well as the implementation of the revitalization program [1, 4, 7]. Over the past two decades, in many Western European countries, compliance with regulations coupled with significant investments in the water industry has significantly reduced the release of organic matter into water bodies [8, 9]. However, despite the efforts to make improve wastewater treatment, at present in many countries final treatment of industrial and household wastewater still occurs in the water body of receivers within the process of natural self-purification [3, 10].

Treated wastewater is a special category of pollution. Due to the trend toward total coverage of industries and settlements with treatment facilities that serve as a barrier between drains and natural reservoirs, the number of such point sources will grow. The runoff from wastewater treatment plants downstream of their inflow significantly alters the quality of the aquatic environment and quantitative characteristics of the population of aquatic organisms in general, and more specifically the assemblages of protozoa [2, 3, 11–18]. However, the relationship between the diversity of organisms and the quality of the environment is not always straightforward. Environmental pollution, especially by organic matter, does not necessarily reduce the diversity of autotrophic and heterotrophic ecosystem components [19]. Accordingly, assessing the impact of such pollution sources and controlling them is an important aspect of protecting the aquatic environment.

To control the degree of pollution of freshwater ecosystems, numerous biotic indices have been developed, in particular, the saprobity index [20], the Extended Biotic Index [21] and some macrophyte indices [22]. Usually, various ecological groups of aquatic organisms are used to assess the anthropic impact, including plankton, benthos, and periphyton [23–25]. For lentic ecosystems–lakes, reservoirs–any of these ecological groups are usually quite informative. In contrast, for rivers, the most informative are the communities of aquatic organisms associated with the benthos [24, 26]. Moreover, under the conditions of flowing water bodies, the communities of the periphyton, which are considered to be systems for the early detection of pollution, can be very informative [23, 25–27].

Protozoa occupy a prominent place in water quality assessment systems due to their wide distribution in all aquatic habitats, as well as a large number of species confined to certain environmental conditions [28–32]. Out of these, ciliated protozoa are most widely used as bioindicators [33–37]. To date, a wide range of information has been accumulated regarding the ecological preferences of a large number of their species, their indicator weights, and saprobic valences have been established [34–38]. As Foissner rightly notes, the organic pollution of running water is underestimated if microorganisms are not included in the quality assessment [39]. The use of protozoa makes it possible to answer the important questions concerning the structural rearrangements in hydrobocenoses, to assess how radical these changes are and how far they spread downstream.

Most species of aquatic organisms, including Rotatoria, Cladocera, Copepoda, and protozoa, disappear or become scarce in the plankton composition in places of municipal wastewater discharge [17]. However, there is an increase in the biomass of benthic organisms [3]. An increase in the organic load (energy subsidies) leads to a change in the trophic structure of the ciliate assemblage formed in the benthic layer. Due to the energy subsidy in the form of sewage, there is a significant decrease in the proportion of algophages and an increase in the proportion of bacteriophages in the assemblage of ciliated protozoa [2]. In a study in the Cuiabá River, Central Brazil, the species composition and abundance of protozoa increased in the areas of the river exposed to pollution where water quality demonstrated higher nutrient concentrations and lower oxygenation levels [18]. This study illustrates that an increase in the diversity of individual taxonomic groups of organisms is not always an indicator of improved habitat and community health.

Of course, because of the characteristics of different rivers, the ratio of river volume to runoff, and the quality of runoff, assessment results can vary significantly and the search for universal indicators becomes more difficult. For example, having studied the diversity of algae and invertebrates using sediment glasses before and after runoff in the Little Miami River in Ohio, Lewis found that the section affected by the runoff maintained a relatively high diversity of organisms and high similarity in species composition with the upstream section [14]. Gücker et al. [3] noted that the problem of studying the impact of streams on water body ecosystems and the search for universal indicators is related to the fact that modern water bodies, due to a variety of allochthonous factors, can often have an impoverished composition of invertebrate communities and discharges can have an impact that is difficult to predict. Nevertheless, even effectively treated wastewater can have significant impacts on the structure and function of stream ecosystems [3].

This research is devoted to the problem of minimizing the labor intensity of assessing the effect of sewage on the assemblage of ciliated protozoa. A number of studies aimed at studying the response of ciliates’ behavioral groups to pollution and the effect of effluents on the ratio of behavioral groups that do not require species identification have been conducted. Efforts were focused on identifying patterns in changes in the ratio of behavioral groups and opportunities to use this ratio as an indicator of wastewater impact on the state of the river ecosystem.

## Materials and methods

**Fig 1:**
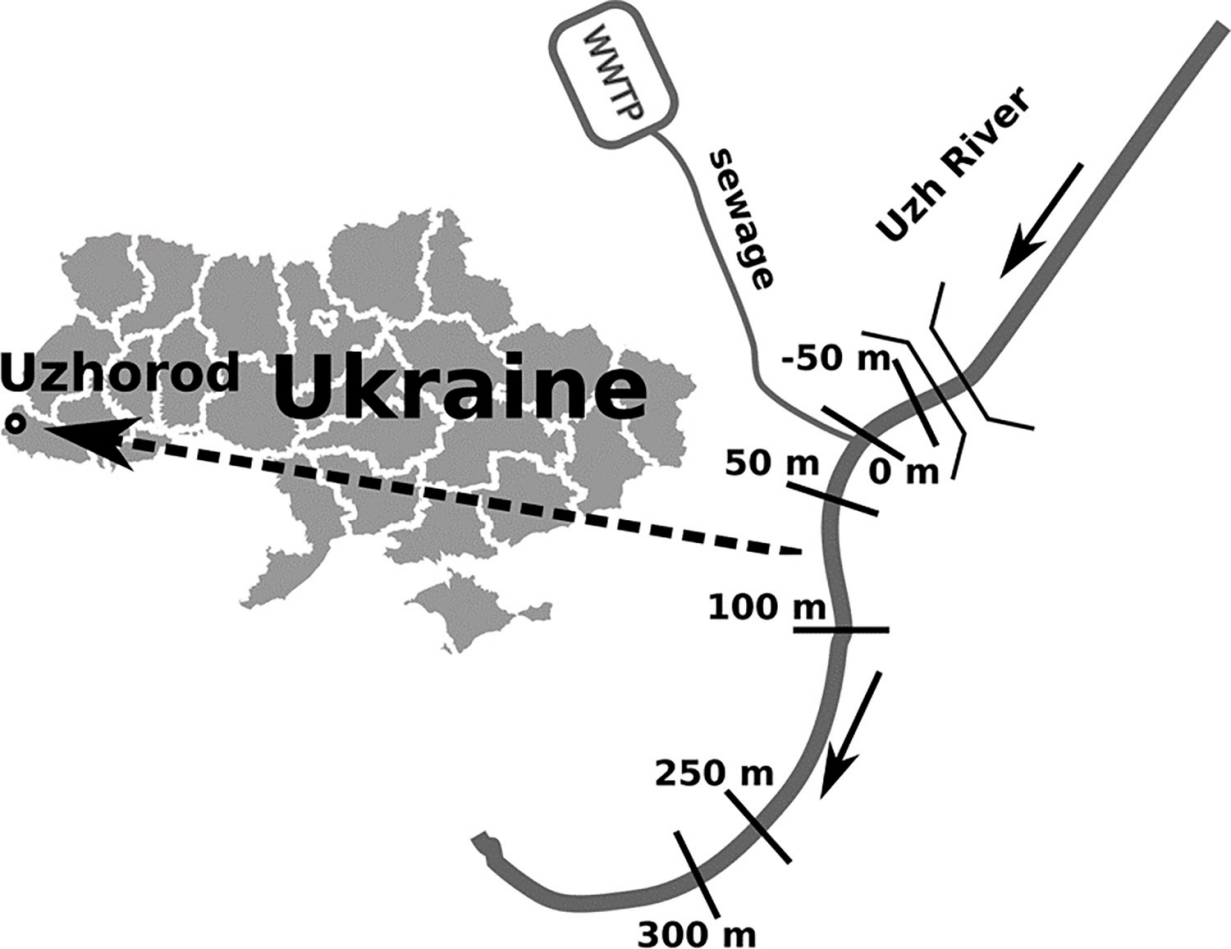
Location of the study area and sampling stations. The arrows along the river show the direction of the current. The dashed arrow indicates the location of the study area on the map. Republished from [40] under a CC BY license, with permission from [SimpleMaps.com Pareto Software, LLC], original copyright [2021].

The samples were taken from the Uzh river on the first and last ten days of each month between February 2016 and February 2018. A total of 236 samples were processed (Fig 1, Table 1).

**Table 1:** Sampling station coordinates on the river Uhz in the area of investigations.

Number of station	Latitude	Longitude
50 м *	48°37’09.32”	22°15’26.77”
**0 м**	48°37’07.30”	22°15’20.55”
50 м**	48°37’05.54”	22°15’18.52”
100 м**	48°37’03.25”	22°15’16.81”
250 м**	48°36’55.63”	22°15’15.65”
300 м**	48°36’52.76”	22°15’16.39”

0 –effluent runoff

*–before runoff

**–after runoff.

River Uzh in the study area has a max depth of 2.7 m, the annual flow at average water availability is about 896 million m^3^, while the average annual volume of runoff from the wastewater treatment plants reaches 18.3 million m^3^, which is 2% of the annual flow of the river. If the volume of runoff from wastewater treatment plants varied insignificantly, the average annual volume of river flow in high-water years reaches 1085 million m^3^ (the share of wastewater inflow– 1.69%), and in low-water years 530 million m^3^, with the share of wastewater volume increases to 3.45%.

The investigated section of the river is located downstream of the city of Uzhgorod and is under the influence of diffuse sources of pollution from the city. The station located 50 m upstream of the drain from the communal wastewater treatment facilities and not experiencing the influence of wastewater was considered by us as a control one. The flow velocity ranges from 1 to 2 m/s. During snowmelt and floods caused by heavy rains, the current speed increases to 4–6 m/s.

At the station 50 m upstream from the runoff, the bottom is rocky and slightly silted. In the section from the inflow of the runoff (station 0) to the point 100 m downstream, the rocky bottom is covered with silt, resembling flakes of activated sludge. At the stations 250 and 300 m below the runoff, the bottom is rocky with an admixture of silted sand, similar in quality to the bottom sediments above the runoff.

The bottom sediment samples from the river were taken using a 100 ml syringe with a 0.5 m long plastic tip and an inlet diameter of 4 mm. Sampling with a syringe allows the most accurate sampling from the required bottom level, excluding the contamination by organisms from the near-surface layers of water. At each point, the samples were taken in triplicate with a volume of 100 ml. At each station, sediment temperature, pH and O_2_ were measured with a HACH HQ40d portable multimeter. The content of nitrogen group compounds and BOD_5_ were also investigated according to the standard methodology. Nitrogen group compounds were determined using a Hach-Lange DR3900 spectrophotometer. Average results by season are presented in Table 2.

**Table 2:** Physical and chemical characteristics of investigated sections of river Uzh (average values period 2017–2018).

Stations	Winter	Spring	Summer	Autumn	
**pH**					
**upstream**	7.47±0,36	7.78±0.22	7.71±0.43	7.51±0.21	
**runoff**	7.26±0.71	7.28±0.62	7.19±0.81	7.25±0.52	
**downstream**	7.45±0.42	7.72±0.35	7.68±0.44	7.43±0.37	
N-NH4 (mgN/dm^3^)					
**upstream**	0.19±0.02	0.04±0.01	0.29±0.05	0.71±0.04	
**runoff**	1.85±0.24	1.99±0.15	1.86±0.15	3.75±0.07	
**downstream**	0.25±0.04	0.45±0.08	0.32±0.07	3.43±0.09	
NO_2_ (mgN/dm^3^)					
**upstream**	0.15±0.03	0.06±0.01	0.08±0.02	0.07±0.01	
**runoff**	0.87±0.05	0.24±0.01	0.63±0.11	1.23±0.10	
**downstream**	0.17±0.03	0.08±0.02	0.12±0.01	1.11±0.03	
N-NO_3_ (mgN/dm^3^)					
**upstream**	1.74±0.23	2.03±0.57	2.03±0.21	5.41±0.64	
**runoff**	14.75±1.21	6.42±0.82	6.73±0.60	11.65±2.52	
**downstream**	2.78±0.63	2.37±0.41	2.74±0.27	6.76±1.71	
**BOD5**					
**upstream**	3.75±0.47	2.72±0.12	5.44±0.91	7.1±1.13	
**runoff**	11.46±0.81	11.95±0.83	14.15±1.20	14.55±1.58	
**downstream**	5.95±0.52	2.92±0.22	5.84±0.60	12.6±1.25	

The bottom samples were taken, delivered to the laboratory, placed in a refrigerator and processed within 24 hours, and this processed according to the procedure described earlier [41–44]. *In vivo* species identification was performed using an Olympus CX41 microscope in transmitted light, as well as dark-field and phase-contrast methods. The population density was estimated by counting in a sample of 25 μl, extracted with a micropipette.

To facilitate the visual identification of ciliates, an oxyprolcellulose solution was added to each sample. This significantly slows down ciliate movement and allows visualization of essential details of the cell structure, the location of the cilia, shape, and position of the macronucleus. If it was impossible to identify ciliates *in vivo*, cells were fixed and stained with methyl green (karyotype) and silver nitrate (argyrome) [45]. Species identification mainly was based on the keys [34–38, 46–52]. Saprobic evaluations were performed using the saprobic values from Sladecek [20] and Foissner et al. [34–38]. The information on the saprobic characteristics of the species is presented in Table 3.

**Table 3:** List of ciliate species and their saprobic parameters (by [20, 37]) identified in the section of the Uzh river.

Species	S	Valences					I	SI
		x	o	b	a	p		
*Acineria uncinata*	a-p			2	4	4	2	**3,2**
*Acineta fluviatilis*								** **
*Amphileptus pleurosigma*	b-a			5	5		3	**2,5**
*Aspidisca cicada*	a-b			4	5	1	2	**2,7**
*Aspidisca lynceus*	b-a		1	4	4	1	1	**2,5**
*Carchesium polypinum*	a			2	7	1	3	**2,9**
*Chilodonella uncinata*	a			2	6	2	3	**3**
*Climacostomum virens*	b			8	2		4	**2,2**
*Coleps elongatus*	a				10		5	**3**
*Colpidium colpoda*	p-i				2	8	4	**3,8**
*Cristigera setosa*								** **
*Dexiostoma campylum*	p-i				1	9	5	**3,9**
*Epistylis chrysemydis*	a			2	6	2	3	**3**
*Epistylis coronata*	a				10		5	**3**
*Epistylis entzii*	a			2	7	1	3	**2,9**
*Epistylis plicatilis*	a-b			3	6	1	3	**2,8**
*Enchelyomorpha vermicularis*	p-m					10	5	**4**
*Euplotes moebiusi*	a			2	7	1	3	**2,9**
*Euplotopsis affinis*	b-a			5	4	1	2	**2,6**
*Frontonia angusta*	b-a			5	5		3	**2,5**
*Frontonia leucas*	b-a		2	3	3	2	1	**2,5**
*Halteria chlorelligera*	o		8	2			4	**1,2**
*Histiobalantium natans*								** **
*Holophrya discolor*	a-b			4	4	2	2	**2,8**
*Holosticha pullaster*	b-a		1	4	4	1	1	**2,5**
*Loxophyllum meleagris*	b			8	2		4	**2,2**
*Mesodinium acarus*	b		2	6	2		3	**2**
*Metopus barbatus*	p-m					10	5	**4**
*Metopus es*	p-m					10	5	**4**
*Opercularia microdiscus*	a				10		5	**3**
*Opercularia coarctata*	a			2	7	1	3	**2,9**
*Oxytricha chlorelligera*	a				10		5	**3**
*Oxytricha setigera*	a-b			4	6		3	**2,6**
*Paramecium bursaria*	b-a			6	3	1	3	**2,5**
*Paramecium caudatum*	p-a				4	6	3	**3,6**
*Paramecium putrinum*	p			1	2	7	3	**3,6**
*Paraurostyla weissei*	a			2	7	1	3	**2,9**
*Plagiocampa rouxi*	a-b			4	6		3	**2,6**
*Pleuronema coronatum*	b			7	3		4	**2,3**
*Pseudovorticella elongata*	b			10			5	**2,3**
*Spirostomum ambiguum*	a			2	6	2	3	**3,0**
*Stentor roeselii*	a-b		1	4	5		2	**2,4**
*Stylonychia mytilus*	a			1	9		5	**2,9**
*Tachysoma pellionellum*	b-a		1	4	4	1	1	**2,5**
*Tetrahymena pyriformis-*complex	p-i				3	7	4	**3,7**
*Tokophrya carchesii*	a			2	7	1	3	**2,9**
*Tokophrya infusionum*	b-a		2	5	3		2	**2,1**
*Tokophrya lemnarum*	a			1	7	2	3	**3,1**
*Tokophrya quadripartita*	a-b			3	5	2	2	**2,9**
*Trachelius ovum*	a-b		1	4	4	1	1	**2,5**
*Trochilioides recta*	a				10		5	**3**
*Trithigmostoma cucullulus*	a-p			2	5	3	2	**3,1**
*Uroleptus piscis*	a			3	7		4	**2,7**
*Uroleptus musculus*	a			1	8	1	4	**3**
*Vorticella aquadulcis-*complex	b-a		2	5	3		2	**2,1**
*Vorticella convallaria-*complex	a		1	2	6	1	2	**2,7**
*Vorticella infusionum-*complex	p-a			1	4	5	2	**3,4**
*Vorticella microstoma-*complex	p-a				5	5	3	**3,5**
*Vorticella picta*	b		2	6	2		3	**2**

Abrevirations: S = saprobity, x = xenosaprob, o = oligosaprob, b = betamesosaprob, a = alphamesosaprob, p = polysaprob, i = isosaprob, m = metasaprob, I = indication weight, SI = saprobity index of concerned species.

The data on ciliated protozoan population abundances were averaged over the seasons. Simpson’s dominance index, Pantele-Buck saprobity index, Shannon’s index and rank-abundance curves were calculated based on averaged data for the entire period of the study. These data on the species abundances were processed using R Environment Version 4.0.3 [53] with the package tidyverse [54]. Shannon’s H and Simpson’s D were calculated using the Biodiversity R package [55]. Plots were produced with the R package ggplot2 [56]. Regression lines in Figs 6 and 7 were smoothed using Locally Weighted Scatterplot Smoothing (LOESS) using provided by ggplot2 option ‘goem_smooth(method = ‘loess’).

## Results

During the study 59 species of ciliated protozoa were identified in the studied section of the river (Table 3).

**Fig 2:**
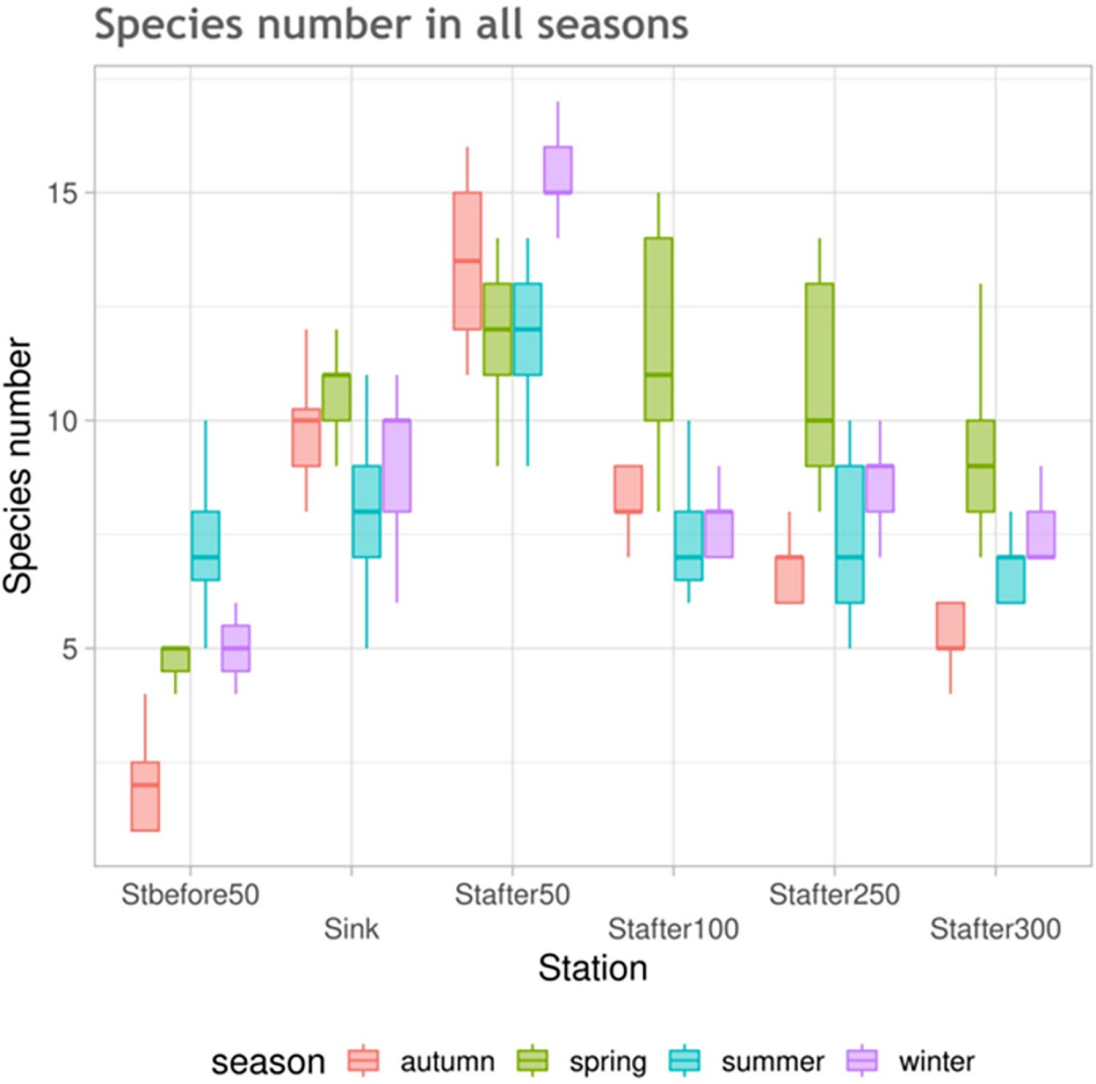
Seasonal changes in the species number by stations in the environmental gradient based on data for the entire study period.

The number of identified species at each station and their seasonal changes are shown in Fig 2.

Organic pollution provoked a noticeable increase in the diversity of ciliated protozoa and this tendency persists during all seasons. To a large extent, this is also due to the appearance on the bottom of typical representatives of periphyton–sessile forms of infusoria, mainly from genera: *Carchesium*, *Opercularia*, *Epistylis* and *Vorticella*. Characteristically, representatives of these genera were practically not registered at the control station. This is a convincing argument in favor of the fact that it is the runoff from the wastewater treatment plant that is the primary cause of the observed phenomenon.

**Fig 3:**
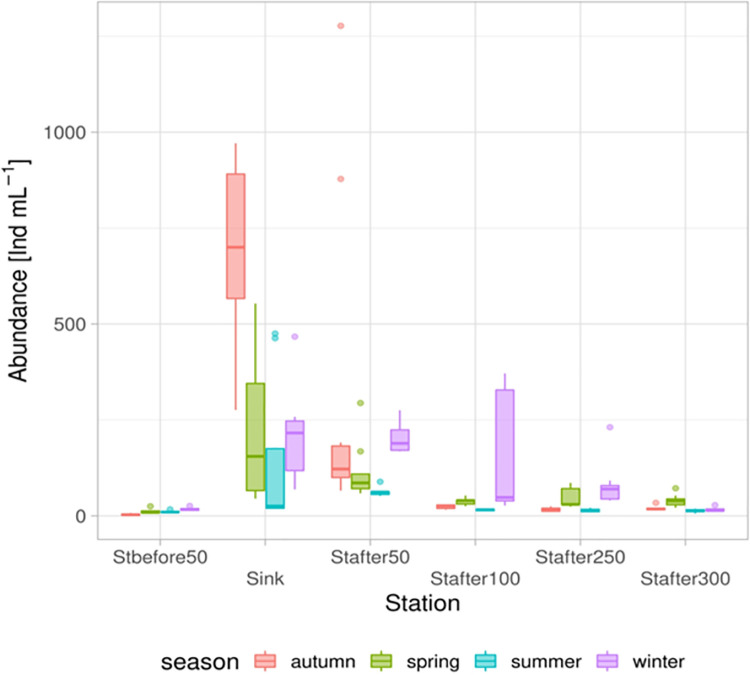
Seasonal changes in the abundance of ciliates by stations in an environmental gradient based on data for the entire study period.

According to the data obtained, it is likely that organic substances coming with wastewater promoted the increased number of species recorded downstream of the runoff when compared to the upstream station. This subsidy also likely responsible for the increased i population density of ciliates; especially in the area up to 50 m below the runoff discharge point (Fig 3). Already at 100 m below the runoff discharge point, the population density decreases significantly. Nevertheless, it remained noticeably higher than at the upstream station located 50 m above the inflow of the treatment plant effluent.

**Fig 4:**
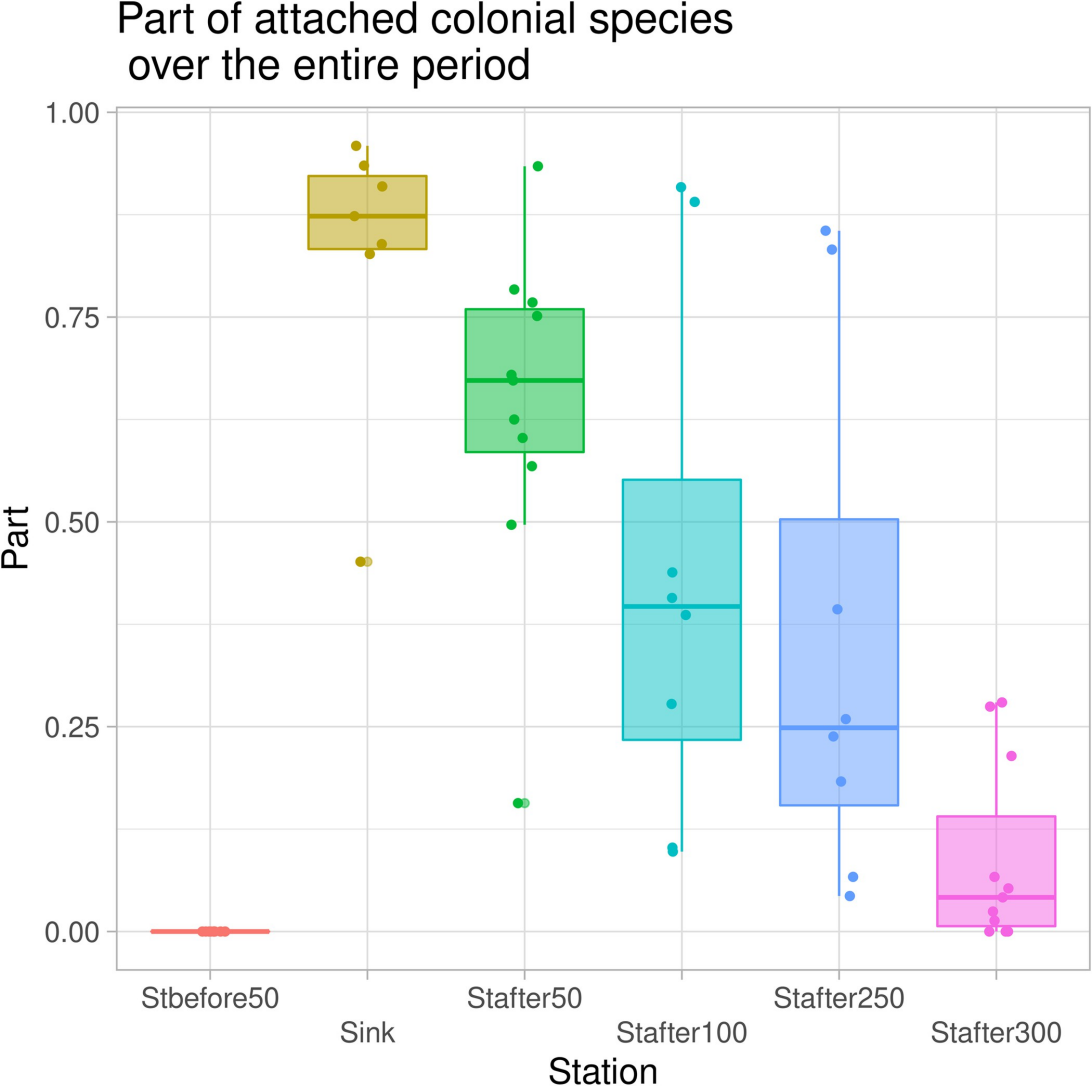
Change in the share of attached forms in the composition of ciliates assemblages at sampling stations based on data for the entire study period.

Representative taxa of sessile forms predominately determine the overall density of assemblages. This was particularly observable at the stations at the site of the influx and up to 100 m downstream of this runoff (Fig 4).

**Fig 5:**
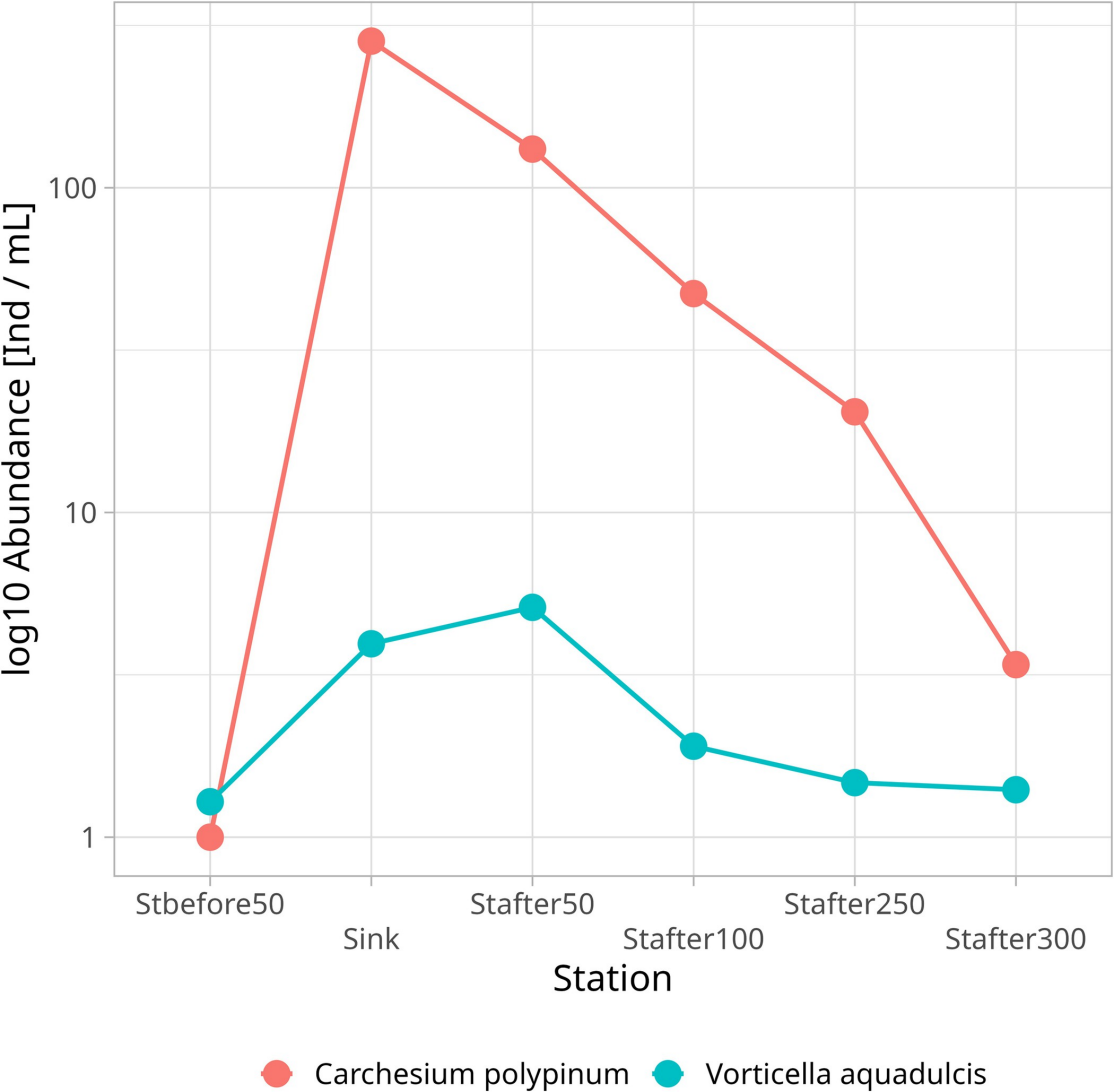
Changes in the abundance of *Carchesium polypinum* and *Vorticella aquadulcis* by stations in an environmental gradient based on data for the entire study period.

Among sessile forms, *Carchesium polypinum* was mainly dominant. All other sessile forms had significantly lower population densities. At the same time, their preferences related to overall environmental quality differed as well. For example, *Carchesium polypinum* reached the maximum population densities in the place of the runoff, yet *Vorticella aquadulcis* had significantly lower population densities, but also reached the peak development at the station 50 below the runoff (i.e., where the pollution levels were markedly lower; Fig 5).

**Fig 6:**
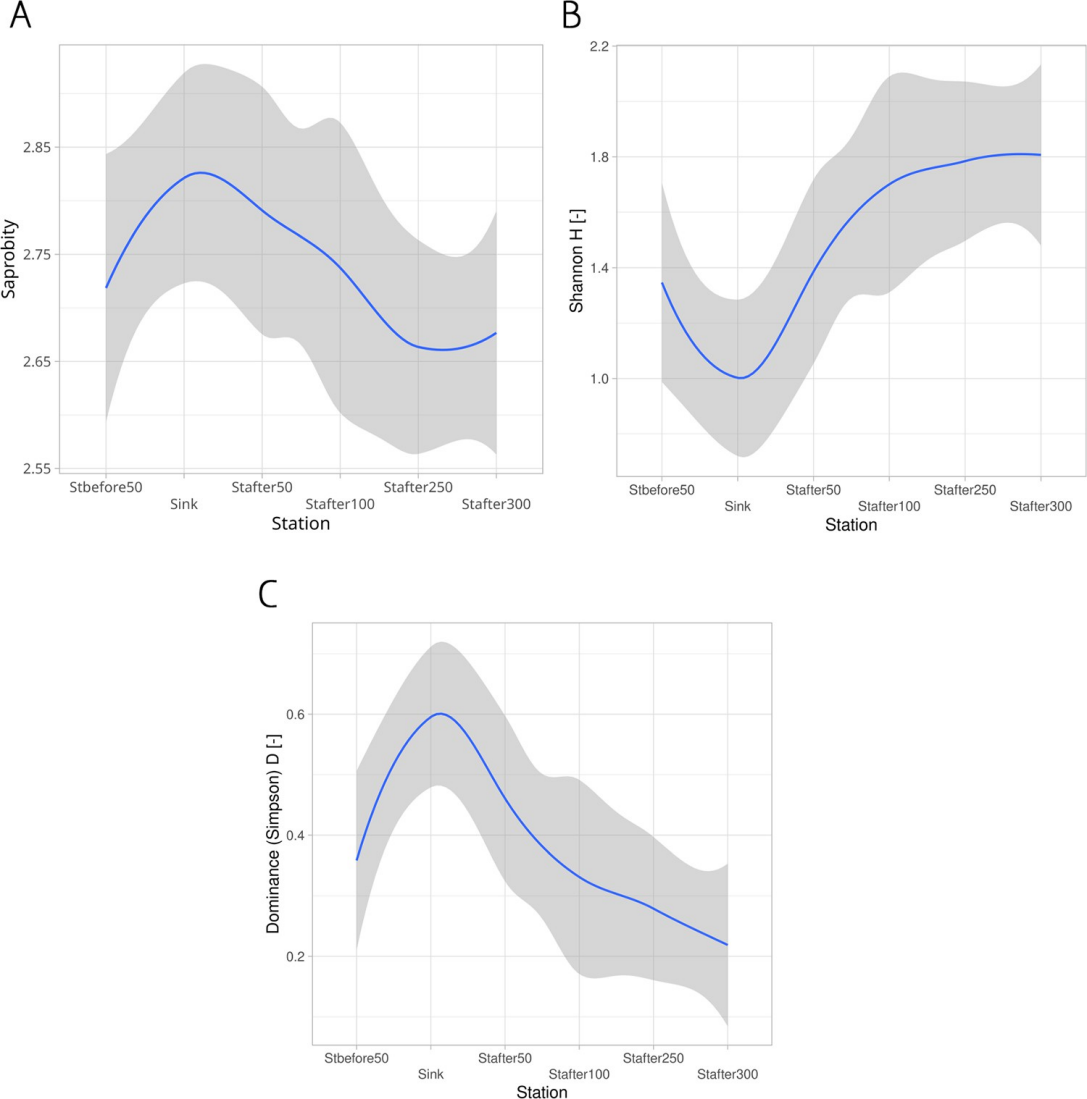
Changes in selected indicators of biodiversity by stations in an environmental gradient based on data for the entire study period. Blue line–loess regression line, gray area– 95% confidence band for loess regression line. (A) Changes in the Pantele-Buck saprobity index. (B) Changes in the Shannon’s index. (C) Changes in the Simpson’s dominance index.

The degree of influence of organic pollution associated with the runoff from treatment facilities was assessed based on the well-known Pantle and Buck saprobity index. The values of the saprobity index over the entire study period are shown in Fig 6A.

The influence of organic pollution in the studied section of the river was most pronounced in the discharge area and in the area up to 50 m below the discharge. Already at 100 meters, the value of the index decreased to the level observed in the section of the river above the confluence of the runoff. Contrary to the expectations, the value of the index did not stabilize here but continued to decline downstream. Evidently, this is a consequence of the ongoing declines in the content of organic matter, and linked, significant improvement in the quality of the environment at these sampling locations.

The level of structural diversity of the assemblage of ciliated protozoa at the studied stations was assessed based on the Shannon’s information index (Fig 6B). The changes in the Shannon’s index were opposite of the changes in the saprobity index. The Shannon’s index reached the level typical for the control station 50 m below the runoff. In the area below 50 meters downstream, a decrease in the level of organic pollution was related to an increase in the Shannon’s index; starting from 100 meters below the runoff, the value of the index stabilized and reached a plateau.

Index of dominance in the assemblage maximized at the discharge point but returned to the level of the control station already at a section of 100 meters below the drain (Fig 6C). Further, at downstream stations the values of the dominance index continued to decline, reaching the values lower than at the control station.

The changes in the diversity indices indicated that the structure of the ciliates population at stations located 100 m downstream from the runoff changed more dramatically than could be expected. According to index estimates, the situation 100 m downstream of the runoff inflow can be considered more favorable than at the control station.

The ecological structure of ciliates assemblages in the studied section of the river was also analyzed. The ratio of groups of species, which we call «behavioral», is an essential characteristic of the assemblage of ciliated protozoa. Species were distinguished in accordance with Madoni [44], who identified the following groups in activated sludge: crawling, free-swimming, and attached. The ratio of these groups can then be used to characterizes trophic conditions of habitat, namely, the availability and localization of main food resource (e.g., bacteria).

**Fig 7:**
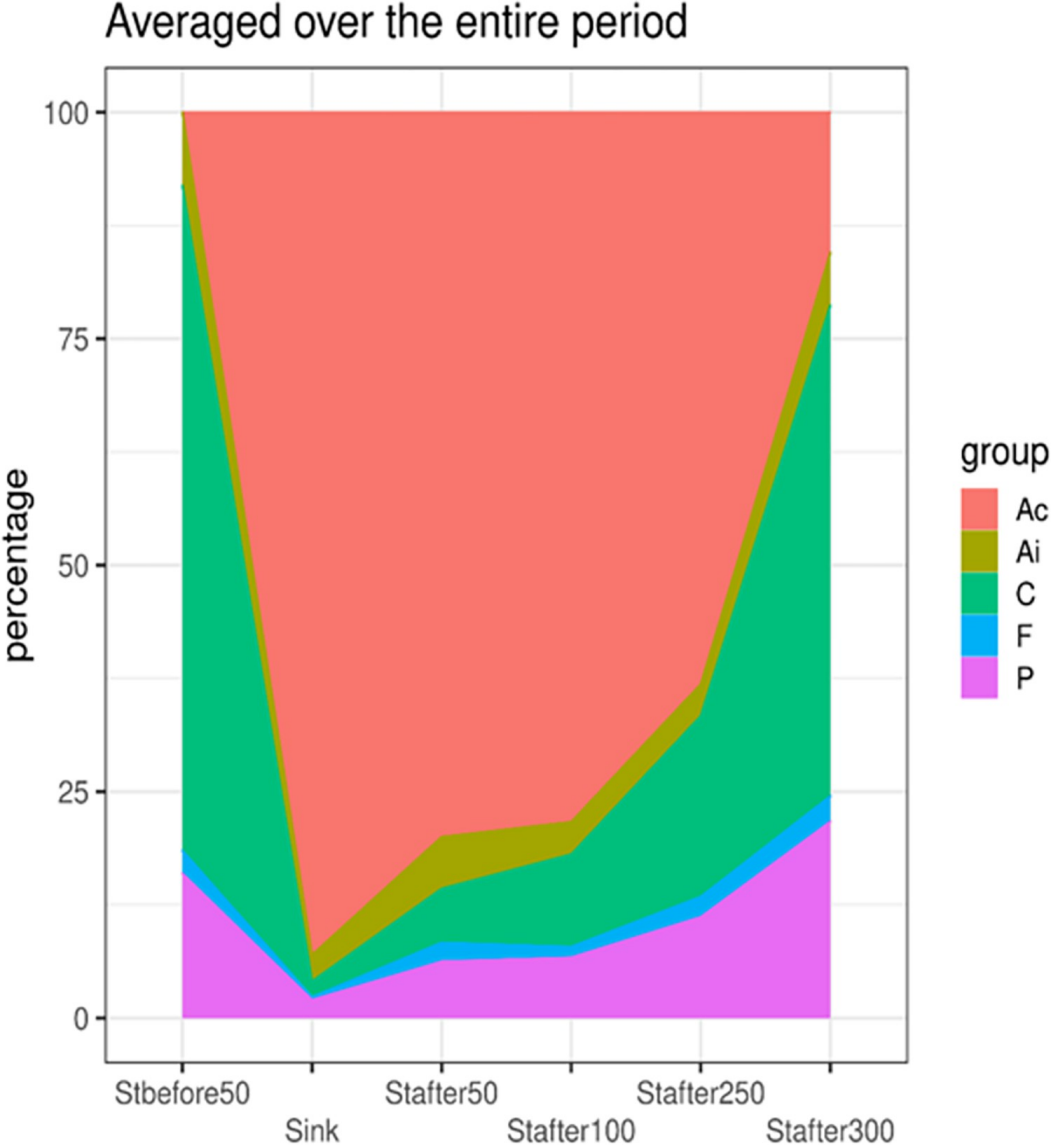
Percentage of behavioral groups and attached colonial species by stations in an environmental gradient based on data for the entire study period. Ac–attached colonial bacterivorous; Ai–attached individual bacterivorous; C–crawling bacterivorous; F–free-swimming bacterivorous; P–predators.

The proportional representation of behavioral groups by stations based on averaged data for the entire study period is shown in Fig 7. Particularly noteworthy is the change in the proportion of sessile forms, primarily of the colonial species of ciliated protozoa. In the area of runoff, the share of colonial forms sharply increases, and further downstream decreased. Although the density of colonial forms even at a distance of 300 m below the runoff remained higher than at the control station, nevertheless, the trend towards a reduction in sessile forms was clear (Fig 7).

**Fig 8:**
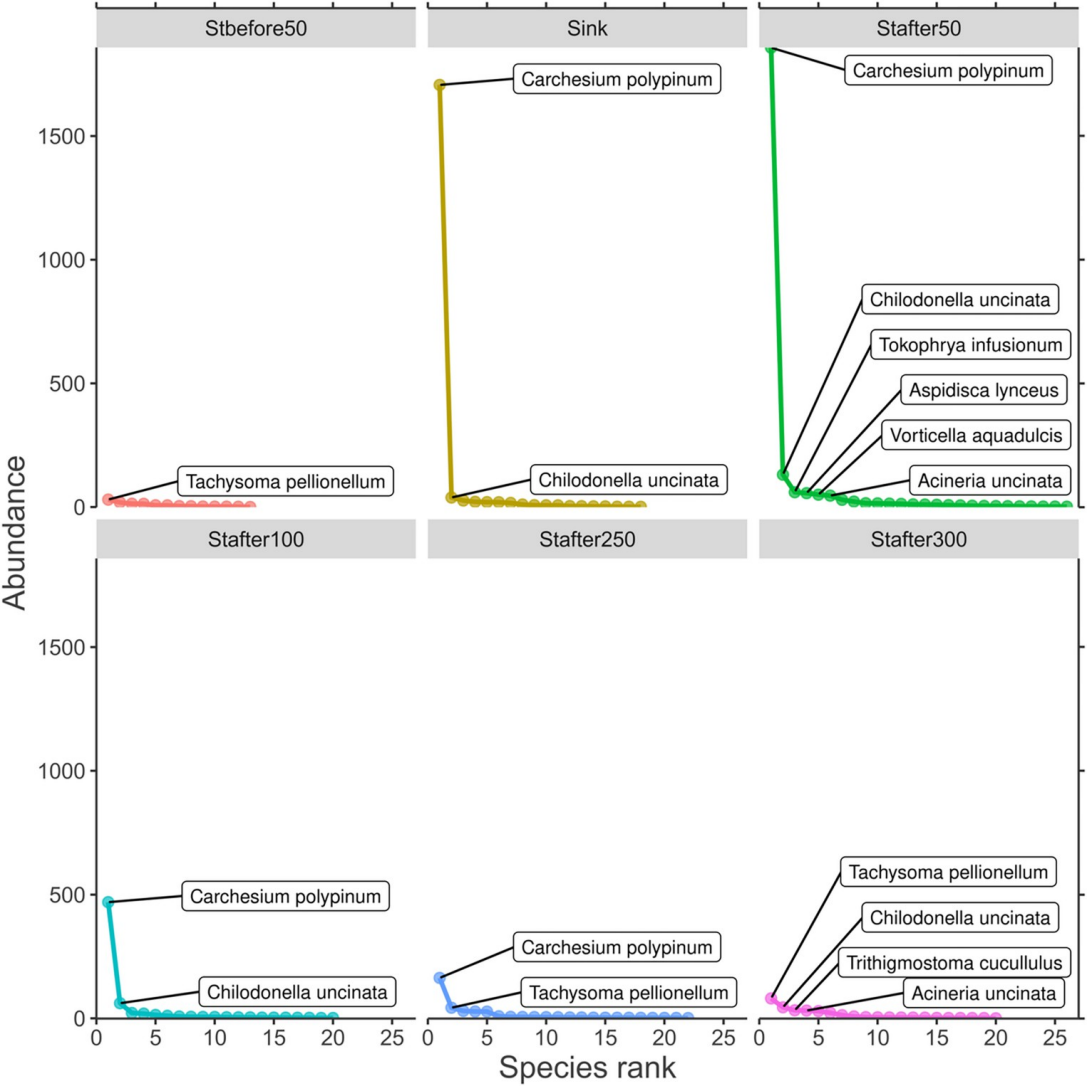
Rank-abundance plots by stations in an environmental gradient based on averaged data for the entire study period.

The recovery of the community structure and the role of sessile forms of ciliates was well demonstrated by the rank-abundance method (Fig 8). The rank structure at the control station showed a relatively low level of dominance; the most abundant species was *Tachysoma pellionellum* a representative of the crawling ecological group. Under the influence of runoff up to 100 m downstream, the rank structure of the population of bottom-dwelling ciliates changed towards a pronounced dominance of the sessile colonial forms, such as *Carchesium polypinum*. Structural recovery was observed at the stations 250 and 300 m below the runoff and *Tachysoma pellionellum* (i.e., the dominant species at the control station) returned to the group of a high-rank species, while *Carchesium polypinum* disappeared from the dominant complex. Similarly, the character of the rank-abundance curves demonstrated a consistent decrease in the role of dominant species and shift to a uniform distribution, as was characteristic of the river at the control station.

Thus, it can be concluded that the changes in the population density of ciliated protozoa and the structure of their assemblages indicated a pronounced improvement of aquatic environment at several hundred meters from the runoff point. Moreover, the structural indices indicated greater than expected changes in the overall quality of the environment.

## Discussion

Ciliates inhabit all possible habitats in almost all types of water bodies. The studied section of the Uzh River is under the noticeable influence of the runoff from the municipal wastewater treatment plant and the composition of the ciliated protozoa had significant similarity with the composition from the reservoir [34, 57]. The ciliates are usually represented by several behavioral groups, which can be easily distinguished morphologically. Usually, ciliates are divided into three morphological groups: crawling, swimming, and attached.

Predominance of the attached forms of ciliates, untypical for bottom sediments was observed in an area up to 100 m below the runoff (Fig 6B). Sessile bacteriovorous ciliates massively developing in this area filter out most of the microflora. Already at 100 m below the runoff, their trophic base is sharply reduced, which naturally leads to a decrease in their numbers. Thus, the influence of the energy subsidy in the form of organic substances coming with wastewater spreads over about 100 m. A sharp decrease in the proportion of sessile forms downstream is the result of their own filtering activity. In this case, the overcompensation effect of energy subsidies supplied by the drain can be observed. Disposal of the energy subsidy in the form of organic-enriched wastewater leads to massive bacterial growth. In turn, most of the bacterial biomass is utilized by sessile bacteriophages, which reach a density unusual for bottom sediments. As a result, there is a kind of hyper-purification of water to a cleaner state than at the control station. Thus, the degree of development of sessile forms in comparison with floating and crawling in bottom sediments can be an indicator of the quality of the environment.

A similar phenomenon is observed in the periphyton in spring. With increased water temperatures, the mass development of bacteria on the dead parts of plants causes similar development of sessile bacteriovorous ciliated protozoa [58]. However, under natural conditions, this situation usually only persists for a limited time and the mass development of sessile ciliates (the so-called spring peak) quickly ends. Two reasons are drive these observation: 1) the exhaustion of the organics resource and 2) the appearance of predatory invertebrates and fish larvae [59–64]. Since the inflow of organic matter with drains is fairly constant, the situation in this section of the river stabilizes and remains unchanged regardless of the season. The systematic flow of wastewater from the treatment plant makes it impossible for most macro- and meso-forms of invertebrates to exist, maximally simplifies the trophic structure of the community living here and creates unprecedented conditions for the development of ciliated protozoa.

As a result, the conditions in the river were similar to those in activated sludge. There was a minimum of predatory forms, and the massive development of bacteriovorous, mainly ciliated protozoa. This population structure promotes the effective filtering of bacteria and water conditioning. In the river 100 m downstream the runoff, the water quality in all respects became higher than at the control station. In our case, the density of ciliates in the control station (above the runoff) was typical for the rivers with mesosaprobic conditions. Here, the benthic community of ciliates was represented by a typical set of species, which includes both filter feeders and predators. Therefore there were no imbalances in the development of any of the trophic groups in this area. We suggest that these conditions reduces the filtration efficiency of bacterial biomass in comparison with the level achieved during the spring peak of self-purification (utilization of organic matter remaining from the fall, mainly dead plant parts) or in an aerotank.

Due to the massive development of sessile filtering ciliated protozoa below the runoff (Fig 3), the situation changes dramatically. Apparently, it is the constantly high number of bacteriovorous ciliates in the area after the runoff, which efficiently utilize the bacterial biomass, which is the reason for the recorded improvement in the values of the indices used in comparison with the values at the control station (Figs 3–5).

Earlier, it was shown that for a satisfactory assessment of the impact of runoff, it is sufficient to analyze the development of ciliate species in the river, which are characteristic of activated sludge [57]. This approach simplifies the analysis and the results obtained in this way are in good agreement with the results obtained on the basis of the analysis of the entire composition of assemblages of ciliated protozoa but this method presupposes a fairly high level of training of the researcher.

Here an even simpler approach is proposed: to assess the quality of the environment and the degree of its restoration based on the ratio of ecological groups of ciliates. As the conducted studies have shown, the analysis of the degree of development of different ecological groups of ciliated protozoa can give results very similar to those obtained on the basis of indices of diversity or saprobity obtained as a result of a full-fledged study of the entire species composition of ciliated protozoa. As can be seen from Fig 6B downstream the runoff, the density of attached forms of ciliated protozoa sharply increases, and after 100 m, a rapid decrease in their density is observed. The probable reasons for this phenomenon were described above Fig 1.

A decrease in the number of attached forms in itself may not necessarily be an indicator of the degree of water purification. These forms, for example, could disappear under the influence of toxic substances. For this reason, the ratio of the abundance of attached forms to the abundance of swimming and crawling forms of ciliated protozoa is likely a more reliable indicator of the completion of the process of utilization of organic pollutants coming with wastewater. The development of swimming and crawling forms at stations below the discharge indicates the absence of toxic effects. As the processes of self-purification are completed and the deficit of the food resource increases, the part of the swimming and crawling forms in the structure of the bottom assemblage of ciliated protozoa increases as well. This allows quantifying the extent of the recovery process. We suggest using the Attached Form Index (AFI) for this purpose:

$$AFI=\frac{A}{C+F}$$ (1)

where A is the number of attached colonial forms, C denotes crawling forms, while F–the free-swimming form. (index alaboratation by Babko R. & Pliashechnik V.).

**Fig 9:**
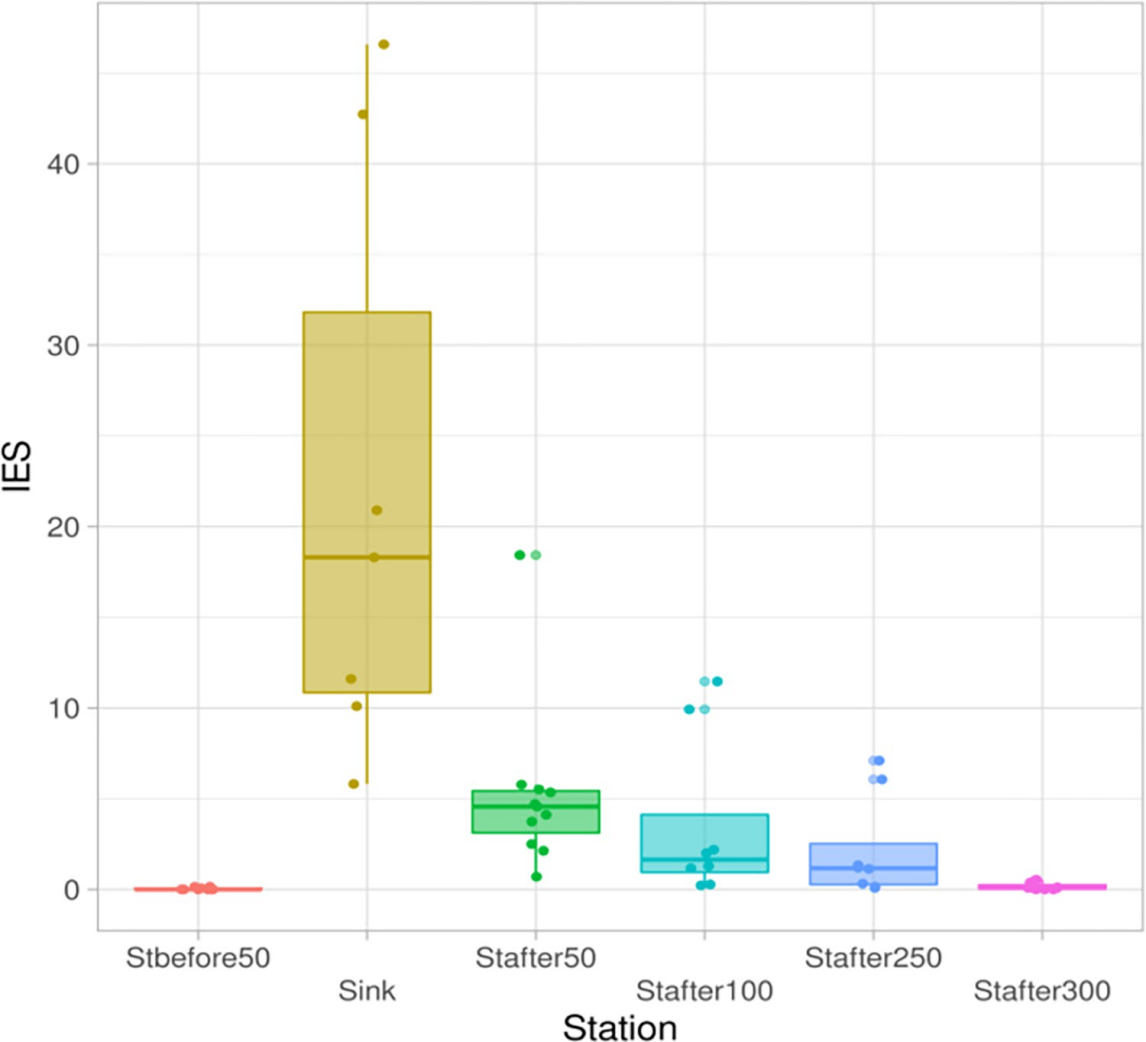
Attached form index (AFI) change by station in the environmental gradient based on data for the entire study period AFI by station.

The AFI values over the seasons give an idea of the change in the ratio of ecological groups in the composition of the ciliated protozoan communities at the stations studied (Fig 9). The index reflects the violation of the natural ratio in the structure of the assemblage of ciliated protozoa under the conditions of bottom sediments. In natural reservoirs, the value of the index tends to zero as a result of the disappearance of attached colonial filtering species. However, under the conditions of constant organic pollution, the index increases by several orders of magnitude, reaching 22 in the runoff. According to AFI, the natural structure of the population of benthic ciliated protozoa is restored only 300 m downstream the runoff, where the index values fall below one: 0.17 (at the control station AFI is equal 0.04).

Thus, the AFI reflects well the processes of restructuring the assemblages of ciliated protozoa under the influence of point sources of pollution, it enables to establish the zone of negative influence of runoff, as well as to assess the degree of restoration of the aquatic ecosystem, the distance at which it occurs.

## Conclusion

The use of the proposed Attached Form Index (AFI) allows drawing the same conclusions regarding the effect of runoff and the distance at which it has a significant impact on the river, as the application of the other assessment methods based on the identification of all species of ciliates from bottom sediments. However, the calculation of the AFI index does not require species-level identifications. Rather, only the abundances of attached, swimming and sessile forms of ciliated protozoa are required. This is a significant advantage of this proposed method, as it can be adopted for use in laboratories at treatment facilities. The use of this express analysis technique also makes it possible to quickly identify the ecological effect of runoff and discharge from municipal treatment facilities, and to pinpoint distances over which these disturbances extend. Overall, the simplicity and efficiency of this method and AFI makes this approach promising for future implementation.

## Supporting information

S1 Data (XLSX)

S2 Data (XLSX)
